# Silicon Application Differentially Modulates Root Morphology and Expression of *PIN* and *YUCCA* Family Genes in Soybean (*Glycine max* L.)

**DOI:** 10.3389/fpls.2022.842832

**Published:** 2022-03-18

**Authors:** Pooja Tripathi, Rupesh Tayade, Bong-Gyu Mun, Byung-Wook Yun, Yoonha Kim

**Affiliations:** Department of Applied Biosciences, Kyungpook National University, Daegu, South Korea

**Keywords:** root morphology, silicon, root length, root surface area, root tip

## Abstract

Silicon (Si) is absorbed and accumulated by some plant species; it has been shown to improve plant growth and performance. The beneficial role of Si in plants is based on the fundamental assumptions, and the biological function of Si is still being researched due to its complex nature, distinctiveness, and interaction. The present study included two distinct experiment sets: a screening test and an advanced test. In the initial examination, we used 21 soybean (*Glycine max* L.) cultivars. Following the evaluation, we chose four cultivars to investigate further. In particular, the positive response cultivars, Taeseon and Geomjeongsaeol, showed a 14% increase in net photosynthesis (*P*_*N*_), and a 19–26% increase in transpiration in Si-treated plants when compared to the control plants. Si-treated Taeseon, Geomjeongsaeol, and Somyongkong, Mallikong cultivars showed significant differences in root morphological traits (RMTs) and root system architecture (RSA) when compared to the control plants. Taeseon and Geomjeongsaeol showed a 26 and 46% increase in total root length (TRL) after Si application, respectively, compared to the control, whereas Mallikong and Somyongkong showed 26 and 20% decrease in TRL after Si treatment, respectively, compared to the control. The Si application enhanced the overall RMTs and RSA traits in Taeseon and Geomjeongsaeol; however, the other two cultivars, Somyongkong and Mallikong, showed a decrease in such RMTs and RATs. Furthermore, to understand the underlying molecular mechanism and the response of various cultivars, we measured the Si content and analyzed the gene expression of genes involved in auxin transport and root formation and development. We showed that the Si content significantly increased in the Si-treated Somyongkong (28%) and Taeseon (30%) compared to the control cultivars. Overall, our results suggested that Si affects root development as well as the genes involved in the auxin synthesis, transport pathway, and modulates root growth leading to cultivar-dependent variation in soybeans.

## Introduction

Silicon (Si) has been identified as a beneficial element in plants that improves plant growth, development, and productivity (Rao et al., 2019). Although Si is an abundant element in the earth, it exists in crystalline and amorphous forms, which plants cannot uptake (Epstein, 1994). The Si regulates nutrient absorption in plants (Tripathi et al., 2014), promotes plant growth and development (Soundararajan et al., 2017), improves yield (Epstein, 1999), enhances biotic and abiotic stress tolerance (Manivannan and Ahn, 2017), and provides resistance to pathogens (Wang et al., 2017). Historically, Japanese farmers pioneered Si usage in agricultural fields by applying slag (calcium silicate as a significant component) into the cultivation of paddy rice (Ali et al., 2008). Over the years, it has been discovered that this substance increases the grain yield while decreasing the incidences of pathogenic and pest attacks (Ma and Yamaji, 2006). Such beneficial behaviors established great excitement among agricultural scientists to further understand the role of Si in various plant growth. Most studies on Si concentrate on its occurrence, soluble forms and bioavailability, uptake and transportation, and stress resistance mechanisms in the plants (Ma and Takahashi, 2002; Mitani and Ma, 2005; Feng et al., 2011). However, many of the studies about the role of Si in plants are based on fundamental assumptions, and because of the intricacy and diverse activity of Si, the biological function is still being researched. Therefore, this has raised much controversy in the scientific community regarding the biological roles of Si and its association with physiological traits. Furthermore, a substantial reassessment of Si role is required, which is crucial for improving the understanding of the biological roles of Si and its supplementation effect on the physiological traits. Moreover, this will aid in guiding future studies and understanding the plant research. Recent studies have discovered that the supplementation of Si altered the stoichiometric ratios of carbon: nitrogen: phosphorus (C: N: P) in wheat (Neu et al., 2017) and rice (Klotzbücher et al., 2018; Deus et al., 2020). Similarly, Si supplementation in sugarcane reduced the C level leading to more absorption of N and P, and increased the biomass production. Besides, Coskun et al. (2019) highlighted and brought together many facts about the favorable Si effects on plant development and yield.

From morpho-physiological perspectives, the root plays a vital role in the Si uptake, transportation, and absorption (Freschet et al., 2021). The absorbed Si can move from the root to the shoot with steam transpiration, especially in the leaf area. Therefore, Si concentration shows massive variation in its assemblage (0.1–10% range of dry weight) (Hodson et al., 2005). However, this also depends on the type and class of plant species (Deshmukh and Bélanger, 2016). Meanwhile, some siliceous plants have a unique Si transportation and genetic absorption mechanism accumulated in different parts of the plants (Deshmukh and Bélanger, 2016). The roots are essential parts of a plant because they not only provide various beneficial effects, such as anchorage to aboveground parts and water uptake from the soil, but are also involved in acquiring nutrients and producing organic acids, such as hormones and amino acids (Yang et al., 2004). Thus, the morphology and the physiology of the roots determine the status of the plant aboveground (Waines and Ehdaie, 2007). For these reasons, the structure and the functioning of the roots are significant in crop productivity, and root research is becoming increasingly relevant in agricultural sectors to increase productivity from several viewpoints. According to Deshmukh et al., 2013, two genes, such as *GmNIP2-1* and *GmNIP2-2*, were involved in the Si transport of soybean root. Furthermore, the discovery of such genes (Deshmukh et al., 2013) has resulted in the classification of soybean as an Si accumulator. Hence, these results assumed that Si is a beneficial element for soybean.

In a previous study by our research group, we observed approximately10–20% increased grain yield in soybean cultivars with the exogenous application of an Si fertilizer for two consecutive years (Tripathi P. et al., 2021). Furthermore, previous studies revealed that the shoot and root morphological traits (RMTs), particularly, the number and size of nodules, were significantly increased by Si fertilizer application. In a previous study, we used an Si fertilizer containing Si and other inorganic ions, such as magnesium (Mg) and calcium (Ca) (composition of Si fertilizer; 25% of SiO_2_, 2% of MgO, and 40% of CaO). The result did not reflect the Si effect alone on root morphology because Mg and Ca are significantly involved in root growth and nodule formation. Therefore, additional research is necessary to discover the effects of Si alone on RMTs. Additionally, Si has a favorable effect on the photosynthetic activity in plant species (Hattori et al., 2003; Maghsoudi et al., 2016; Kleiber et al., 2020; Verma et al., 2020). However, limited information or studies have reported the effect of Si on RMTs and photosynthesis efficiency in soybean plants. For this reason, sodium metasilicate (Na_2_SiO_3_) was used to identify only the Si effects on various RMTs and photosynthesis-related parameters of soybean cultivars.

In this study, we performed a series of experiments in a greenhouse to investigate the impact of Si application on RMTs, RSA traits, and photosynthesis-related parameters of 21 soybean cultivars. The Si application showed a diverse response of RMTs, RSA traits, and photosynthesis-related parameters among the tested cultivars. Additionally, the expression levels of genes, which were known to be involved in auxin transport and root formation and development showed differential transcript levels in contrasting cultivars. This is the first report on the differential cultivar-dependent effect of Si on soybean root and shoot traits.

## Materials and Methods

To identify only the Si effects on soybean root growth and development, we performed the experiment in two parts. Experiment I (EP-I) consisted of screening of contrasting soybean cultivars according to Si application. In experiment II (EP-II), we conducted an advanced investigation of the morphological, physiological, and genetic characters in the selected soybean cultivars.

### Experiment I: Evaluation of Silicon Effect on Various Soybean Cultivars

#### Plant Materials and Growth Conditions for Experiment I

To select the contrasting root phenotypes after Si application, we used 21 soybean cultivars as plant materials (Supplementary Table 1). The seeds were sown in polyvinyl chloride (PVC) pipes [16.5 cm (diameter) × 50 cm (height)] containing horticulture soil without added fertilizer (Tobirang, Baekkwang Fertility, South Korea). The pots were placed in a greenhouse at the research center of Kyungpook National University, Daegu, South Korea. The experiment was conducted in a completely randomized design in a fixed position arrangement. For each cultivar, two seeds were sown per pot, with three replications (*n* = 5). Seedlings were grown under regulated photoperiod and temperature (photoperiod: 14 h daytime, temperature: 28°C ± 2°C). When the plants reached the vegetative growth stage 1 (V1), we randomly divided them into two groups: control and treatment plants. Then, we applied Si in the form of (2 mM) sodium metasilicate (Na_2_SiO_3_, Sigma-Aldrich, United States) solution (100 ml) to the treatment plants by soil drenching and foliar spraying for 10 days. Similarly, the control plants were irrigated with 100 ml of water by soil drenching and foliar spraying with only water for 10 days. For the root phenotype investigation, the samples were collected at the end of the experiment (10 days after treatment; DAT).

#### Determination of Root Morphological Traits in 21 Soybean Cultivars

We removed the entire soil from the PVC pipes and separated the root samples. Then, the roots were gently washed under tap water and were kept in plastic bags containing 10—15 ml of water to preserve moisture in the root samples. We captured root images using a scanner (Expression 12000XL, Epson, Japan). The collected root samples were placed in a transparent tray (30 cm long × 20 cm wide, it was made with the acryl) containing clean water for scanning (Supplementary Figure 1). Then, we saved the scanned root images as 2D and analyzed them using WinRHIZO Pro software (Regent Instruments Inc., Canada).

#### Experiment II: Effects of Silicon on Shoot and Root Morphological Traits in Selected Soybean Cultivars

Based on the initial RMT screening result of soybeans, four soybean cultivars (Geomjeongseol, Taeseon, Somyongkong, and Mallikong) were selected. In EP-II, the selected soybean cultivars were used as plant materials and various evaluations related to physiological and morphological traits of shoot and root after Si application were conducted. Specifically, we analyzed RSA traits, such as the number of tips (NTs) and the number of forks (NFs), to confirm the effects of Si on RMT and RSA.

#### Plant Materials and Growth Conditions for Experiment II

Four cultivars were grown in PVC pipes [16.5 cm (diameter) × 50 cm (height)] containing sandy soil. The same planting method with EP-I was employed in EP-II. When the plants reached the V1 growth stage, we randomly divided them into two groups: control and treatment (soil + leaf) plants. Si application and other evaluation methods were conducted in the same manner as in EP-I. Si was applied to the plants (soil drenching + foliar) for 7 days. EP-II employed the same Si concentration as EP-I. The shoot and root phenotype data were collected at the end of the experiment (7DAT).

#### Determination of Root and Shoot Phenotypes

To identify the Si effect on shoot phenotype, we used a 2D shoot image in which imagery samples were captured from the control and treatment groups on 7DAT. The image was captured using an RGB camera (mirrorless M100, Canon, Japan) (Supplementary Table 2). We analyzed the collected shoot images using the WinDIAS image analysis system (Delta-T devices, United Kingdom). Overall, the root samples were collected and analyzed following a previously mentioned protocol in EP-I.

#### Measurement of Photosynthetic Parameters

Photosynthesis-related parameters were analyzed on alternate days after the Si application. A portable photosynthesis meter (LI-6800, LI-COR Inc., Nebraska, United States) was used for measuring the photosynthetic attributes. We selected a fully expanded second trifoliate leaf to obtain uniformity in data for examining photosynthesis. For precise measurement, the samples were handled with utmost care and proper acclimation time was followed. During the measurement period, the airflow was continuously supplied at 700 μmol s^–1^ to the leaf chamber. The concentration of CO_2_ in the leaf chamber was maintained at 400 μmol mol^–1^ during detection, and water vapor pressure was adjusted to 2.00 ± 0.15 kPa during the entire data collection period. The temperature of the leaf chamber was fixed at 28°C, which was set based on the temperature of the greenhouse. In our experiment, we used LED light as a source of light; thus the light intensity of the leaf chamber was set to 1,000 μmol m^–1^ s^–1^ and the ratio of the red and blue light was set as r90:b10 based on the manufacturer’s recommendation. After completing the environmental setting, we examined whether all the set values were working correctly, and then we began the measurement. We carefully monitored the net photosynthesis and stomatal conductance curves to collect uniform data. When both values showed stability, we pressed the measurement button. The parameters, net photosynthesis (*P*_*N*_), transpiration rate (*E*), and stomatal conductance of water vapor (*g*_*sw*_), were used as growth indicators. Data were collected for control and treatment (soil + leaf) plants, with three replications (*n* = 5).

### Determination of Silicon Contents

We used similar growth methods as EP-I and EP-II in a growth chamber. When the plants reached V2, we applied 100 ml of 2 mM of Si to each soybean plant for 7 days and the same volume of water for the control group. After the treatment period, the samples of the shoot were harvested for Si uptake analysis. The plant samples were thoroughly washed with double-distilled water to prevent contamination. Then, we soaked the samples in 0.5 M of HCl for 20 s and rinsed them with double-distilled water (Kaya et al., 2009). Next, the samples were dried in an oven at 80°C for 72 h. The samples were placed in liquid nitrogen and kept in a deep-freezer (-80°C). Detailed methods for determining the Si contents of plants have been previously reported by Kim et al. (2014). Samples were freeze-dried (-50°C) and then ground into a fine powder. Then, 1 g of the dried sample was digested in 5 mL of a tertiary mixture of HNO_3_, H_2_SO_4_, and HClO_4_ [10: 1: 4 (v/v/v)]. The concentration of Si was determined using inductively coupled plasma (ICP, Optima 7900DV, Perkin-Elmer, United States). Data were statistically analyzed using GraphPad Prism Software (Version 5.01) to evaluate quantitative differences.

### Quantitative Real-Time PCR Analysis

To understand the response of various cultivars after Si application at a molecular level, two soybean cultivars, Taeseon (positive response cultivar) and Somyongkong (negative response cultivar), were selected for gene expression analysis. Further, soybean plants were grown in a growth chamber with 14 h photoperiod, 70% relative humidity (RH), the daytime temperature of 28 ± 2°C, the light intensity of 1,000 μmol m^–1^ s^–1^, and the ratio of red and blue light was set as r90: b10. The Si solution was applied using the above-mentioned method for 7 days. Root samples were collected at 0, 3, 6, and 12 h after Si application. We extracted total RNA using the Trizol method (Invitrogen, Carlsbad, CA, United States), followed by ethanol precipitation and purification before complementary DNA (cDNA) synthesis. Further, 1-μg extracted RNA was used for cDNA) synthesis using HiGene Real-Time (RT) kit (BIOFACT, Daejeon, Korea), according to the manufacturer’s instructions. Quantitative real-time PCR (qRT-PCR)-based gene expression was performed using a two-step PCR program. Briefly, each reaction volume contained 20 μl of PCR mix, containing 2times RT PCR master mix, including SYBR Green 1 (BIOFACT, Daejeon, Korea), 10 pmol/μl of each primer (Supplementary Table 3), and 1-μl cDNA, and we adjusted the final volume to 20 μl by adding nuclease-free water. Sample reactions were conducted using a PCREco real-time PCR system (Illumina, San Diego, CA, United States) with a PCR program starting with an initial denaturation at 95°C for 15 min followed by denaturation at 95°C for 15 s and annealing and extension for 40 cycles at 60°C for 30 s.

### Statistical Analysis

The experimental design was randomized with five replications. To determine statistical significance, we analyzed the variance (ANOVA) (SAS release 9.4; SAS, Gary, NC, United States). Similarly, Student’s *t*-test was conducted in R studio to determine whether the means of the two treatments differed significantly from each other at *P* ≤ 0.05, *P* ≤ 0.01, and *P* ≤ 0.0001 levels. A correlation test (Pearson’s r) was conducted to measure the linear correlation among roots and shoot parameters. The figures, except for correlation data, were made with Microsoft Excel (2013) to evaluate the root and shoot traits.

## Results

### Experiment I: Phenotypic Variation in Root Traits of 21 Soybean Cultivars

**FIGURE 1:**
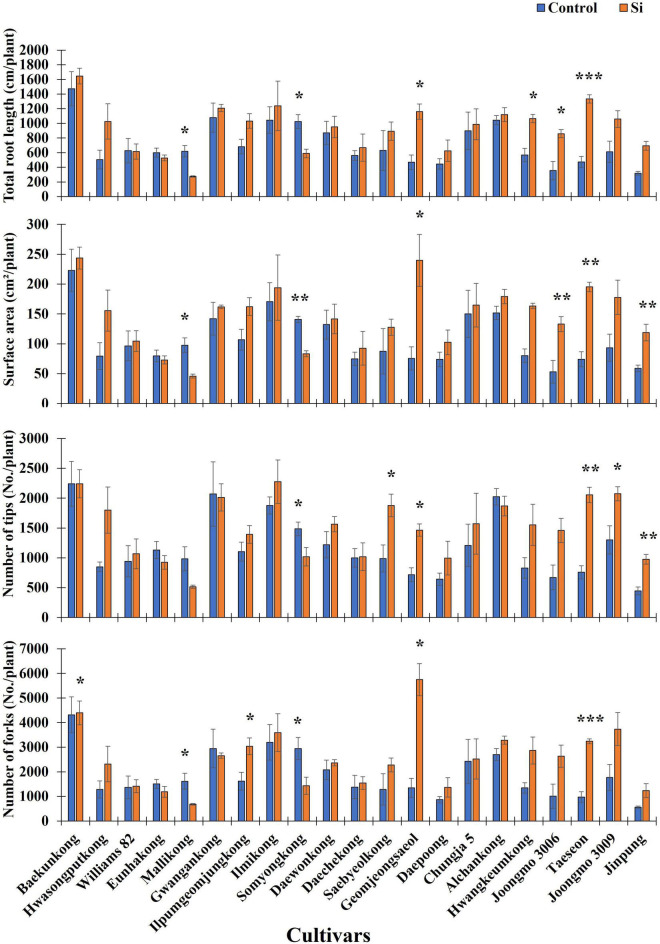
Silicon (Si) effect on total root length (TRL), surface area (SA), number of tips (NTs), and number of forks (NFs) in 21 soybean cultivars. The data were collected 10 days after Si treatment (DAT), and the blue and orange bars represent the control and Si treatment, respectively. In the figure, the vertical bar means average, and error bars indicate standard error. The experiment was conducted by three replications (*n* = *3*). Error bars indicate standard error, and the asterisks indicate significant difference using the Student’s *t*-test (*0.01 < *P* < 0.05; **0.001 < *P* < 0.01; ***P < 0.0001).

According to a previous study, Si fertilizer application induced an increase in RMT, such as total root length (TRL), surface area (SA), NT, NF, and nodule size and number (Ali et al., 2008; Chung et al., 2020; Tripathi D. K. et al., 2021). Thus, to identify only the Si effects on soybean root, we analyzed RMT with the imagery data, and then an ANOVA test was conducted. According to Table 1, TRL, SA, NT, and NF showed significant differences between cultivars and treatments (Table 1). According to Figure 1, except for Mallikong and Somyongkong, enhanced RMT was observed in most Si-treated soybean cultivars compared to the control. Although most soybean cultivars showed an increased tendency, each root trait showed different results. In the case of TRL, Geomjeongsaeol, Hwangkeumkong, Joongmo 3006, Taeseon, and Jinpung showed 148, 88, 142, 182, 73, and 118% increase after Si treatment, respectively, compared to the control (Figure 1). Similarly, Geomjeongsaeol, Joongmo 3006, Taeseon, and Jinpung exhibited significantly increased SA with 218, 150, 163, and 102% in Si application, respectively, compared to the control. Conversely, NT and NF showed dissimilar inclination with TRL and SA. In NT, Saebyeolkong, Geomjeongsaeol, Taeseon, Joongmo 3009, and Jinpung recorded 90, 104, 171, 59, and 118% significant increase, respectively, in Si treatment than the control plants, whereas the NF observed that Ilpumgeomjungkong, Geomjeongsaeol, and Taeseon showed 88, 327, and 233% significant increase after Si supplementation, respectively, compared to the control (Figure 1). Among the four major RMT, the cultivars, Geomjeongsaeol and Taeseon showed constant increased RMT in Si application, while Mallikong and Somyongkong revealed decreased RMT in Si treatment. Thus, we selected four cultivars for further investigation to discover different Si effects on soybean roots.

**TABLE 1:** Analysis of variance (ANOVA) of the shoot and root parameters among 21 soybean cultivars.

Parameters	Source	DF	Type III SS	Mean Square	*F* Value	Pr > *F*
TRL	Variety	20	9,421,797.253	471,089.863	7.4	< 0.0001
	Treatment	1	1,302,646.499	1,302,646.499	20.46	< 0.0001
	Replication	2	3,386,701	1,693,351	37.64	0.00000
	Variety × Treatment	20	2,934,563.815	146,728.191	2.3	0.0044
SA	Variety	20	215,301.6091	10,765.0805	6.71	< 0.0001
	Treatment	1	36,522.8644	36,522.8644	22.75	< 0.0001
	Replication	2	83,391	41,695	33.811	0.0000
	Variety × Treatment	20	84,804.7524	4240.2376	2.64	0.0011
NT	Variety	20	27,527,010.63	1,376,350.53	8.55	< 0.0001
	Treatment	1	3,222,316.61	3,222,316.61	20.02	< 0.0001
	Replication	2	6,196,993	3,098,497	21.117	0.0000
	Variety × Treatment	20	7,934,895.19	396,744.76	2.47	0.0023
NF	Variety	20	104,797,156.8	5,239,857.8	8.15	< 0.0001
	Treatment	1	11,038,244	11,038,244	17.16	< 0.0001
	Replication	2	36,787,852	18,393,926	29.047	0.00000
	Variety*Treatment	20	47,925,358	2,396,267.9	3.73	< 0.0001

*Total root length (TRL), surface area (SA), number of tips (NTs), and number of forks (NFs).*

### Phenotypic Variation in the Shoot and Root Traits of Four Soybean Cultivars

**FIGURE 2:**
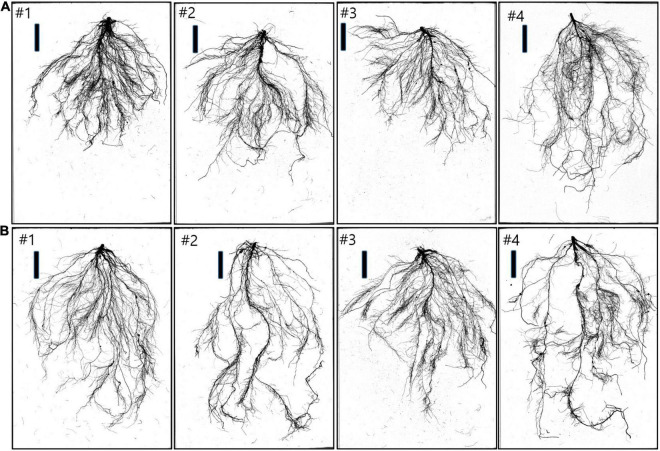
Example of four soybean cultivars with root systems’ morphology 7 days after Si treatment. **(A)** Control root samples of cultivars (left to right): #1 (Geomjeongsaeol), #2 (Taeseon), #3 (Mallikong), and #4 (Somyongkong). Bar = 10 cm. **(B)** Si-treated root samples of cultivars (left to right): #1 (Geomjeongsaeol), #2 (Taeseon), #3 (Mallikong), and #4 (Somyongkong). Bar = 10 cm.

Four soybean cultivars (Geomjeongsaeol, Taeseon, Somyongkong, and Mallikong) were selected based on the initial EP I experiment. Among the four soybeans, Geomjeongsaeol and Taeseon showed significantly increased RMT on Si application, whereas the other two cultivars, Somyongkong and Mallikong, showed reduced RMT after Si treatment compared to the control plant. Figure 2 represents the morphologic variation of the control and Si-treated plants (7DAT) in the root system of four cultivars. According to ANOVA, except for MTL, most shoot and root traits showed significant differences between the four cultivars (Table 2). However, only two traits, such as shoot area and *g*_*sw*_, revealed significant differences within the treatments (Table 2). In the case of the interaction between variety and treatment, we observed statistical differences for several shoot and root traits, such as shoot area, *P*_*N*_, *E*, *g*_*sw*_, TRL, SA, average diameter (AD), NT, and NF (Table 2).

**TABLE 2:** Analysis of variance (ANOVA) of the various shoot and root traits among soybean cultivars for two treatments.

Parameters	Source	DF	Type III SS	Mean Square	*F*-value	Pr > *F*
Shoot area	Variety	3	190,393.6357	63,464.5452	18.39	< 0.0001
	Treatment	1	25,396.4951	25,396.4951	7.36	0.0073
	Replication	4	35,142	8786	3.1632	0.0156
	Variety × Treatment	3	25,319.326	8439.7753	2.45	0.0653
*P*_*N*_	Variety	3	1,786.272935	595.424312	57.04	< 0.0001
	Treatment	1	0.000536	0.000536	0	0.9943
	Replication	4	50	12.5	0.934	0.4435
	Variety × Treatment	3	202.35407	67.451357	6.46	0.0004
*E*	Variety	3	0.00042093	0.00014031	46.59	< 0.0001
	Treatment	1	0.00001105	0.00001105	3.67	0.0572
	Replication	4	0.00001	0.000002	0.524	0.718
	Variety × Treatment	3	0.00003314	0.00001105	3.67	0.0135
*g*_*sw*_	Variety	3	3.23059549	1.07686516	31.43	< 0.0001
	Treatment	1	0.16047648	0.16047648	4.68	0.0319
	Replication	4	0.000003	0.0000008	0.171	0.9531
	Variety × Treatment	3	0.21392242	0.07130747	2.08	0.1046
TRL	Variety	3	33,655,027.95	11,218,342.65	71.3	< 0.0001
	Treatment	1	309,518.19	309,518.19	1.97	0.1624
	Replication	4	194,740	48,685	0.3044	0.8747
	Variety × Treatment	3	8,481,657.77	2,827,219.26	17.97	< 0.0001
SA	Variety	3	603,909.1662	201,303.0554	60.43	< 0.0001
	Treatment	1	7,904.9833	7,904.9833	2.37	0.1251
	Replication	4	2,490	623	0.1786	0.9491
	Variety × Treatment	3	177,455.7189	59,151.9063	17.76	< 0.0001
AD	Variety	3	0.06082022	0.02027341	17.14	< 0.0001
	Treatment	1	0.00090738	0.00090738	0.77	0.3822
	Replication	4	0.006675	0.001669	1.6012	0.1765
	Variety × Treatment	3	0.01237717	0.00412572	3.49	0.0168
NT	Variety	3	56,941,452.3	18,980,484.1	49.29	< 0.0001
	Treatment	1	4,617.61	4,617.61	0.01	0.9129
	Replication	4	85,353	21,338	0.0552	0.9943
	Variety × Treatment	3	20,045,287.37	6,681,762.46	17.35	< 0.0001
NF	Variety	3	1,267,728,822	422,576,274	74.89	< 0.0001
	Treatment	1	15,957,860	15,957,860	2.83	0.0942
	Replication	4	12,685,242	3171,310	0.5272	0.7159
	Variety × Treatment	3	223,722,299	74,574,100	13.22	< 0.0001

*Net photosynthesis (P_N_), transpiration rate (E), stomatal conductance of water vapor (g_sw_), total root length (TRL), surface area (SA), the average diameter (AD), number of tips (NTs), number of forks (NFs), link average diameter (LAD), main total length (MTL) and lateral total length (LTL).*

#### Experiment II: Application of Silicon and Phenological Changes in the Shoot Area

**FIGURE 3:**
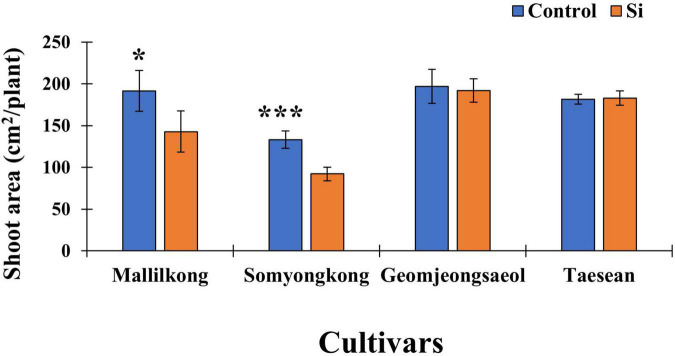
Silicon effect on the shoot area in selected soybean cultivars. The data were collected at different periods, and the blue and orange bars represent control and Si treatment, respectively. In the figure, the vertical bar means average and error bars indicate standard error. The experiment was conducted by five replications (*n* = *5*). Error bars indicate standard error, and the asterisks indicate significant difference using the Student’s *t*-test (*0.01 < *P* < 0.05; ***P < 0.0001).

According to Figure 3, the shoot area showed an interesting result. Mallilkong (25%) and Somyongkong (31%) had significantly reduced the shoot area in the Si treated plants compared to the control; however, the other two cultivars, Geomjeongsaeol and Taeseon, showed no difference between the control and the Si treated plants (Figure 3).

#### Experiment II: Photosynthesis and Related Parameters

**FIGURE 4:**
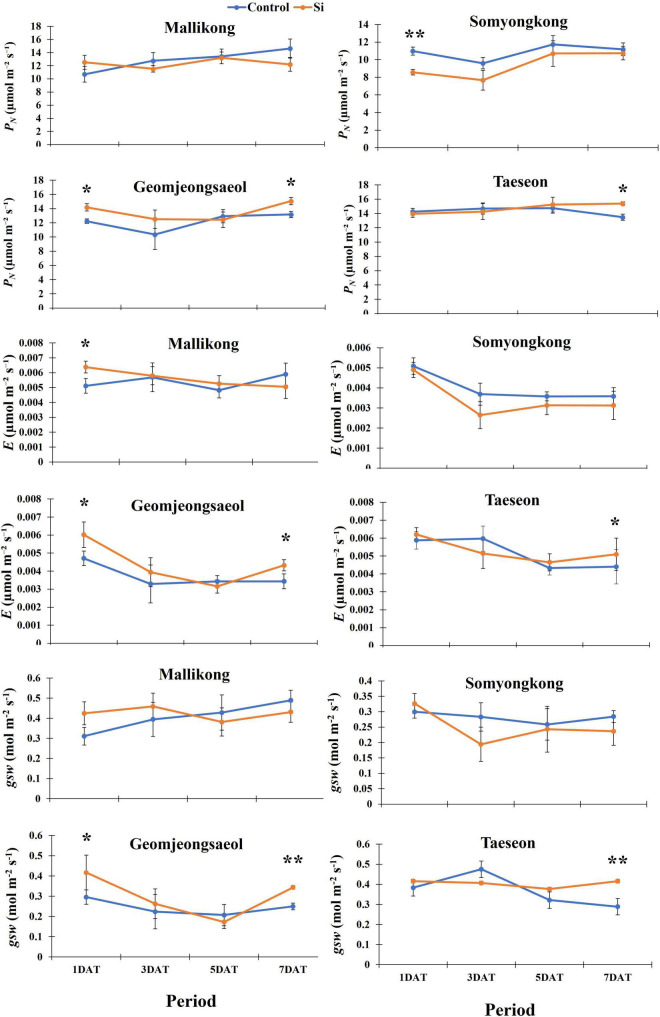
Silicon effect on net photosynthesis (*P*_*N*_), transpiration rate (*E*), and stomatal conductance of water vapor (*g*_*sw*_) in selected soybean cultivars. The data were collected at different periods, and the blue and orange lines represent control and Si treatment, respectively. In the figure, each circle means average, and error bars in the circle indicate standard error. The experiment was conducted by five replications (*n* = *5*). Error bars indicate standard error, and the asterisks indicate significant difference using the Student’s *t*-test (*0.01 < *P* < 0.05; **0.001 < *P* < 0.01).

To analyze the effects of Si on photosynthesis, we measured *P*_*N*_ and the related parameters. According to Figure 4, contrasting soybean cultivars showed a definite difference in *P*_*N*_ and its parameters. In the case of *P*_*N*_, Mallikong and Somyongkong showed no difference between the control and the Si-treated plants (Figure 4). Although *P*_*N*_ was reduced in Si-tr*e*ated Somyongkong at 1DAT, it was reversed at 3DAT (Figure 4). Conversely, Geomjeongsaeol and Taeseon showed a 14% increase in *P*_*N*_ in Si-treated soybean plants over non Si-treated soybean plants at 7DAT, even though other periods did not show any difference between the control and the treated plants (Figure 4). Furthermore, *E* showed a similar tendency with *P*_*N*_. Except for Mallikong at 1DAT, Mallikong and Somyongkong showed no difference between the Si treated and control, while increased *E* was observed in Si-treated Geomjeongsaeol and Taeseon compared to the control plants (Figure 4). Particularly, Geomjeongsaeol showed an increased transpiration rate at 1DAT (28%) and 7DAT (26%) (Figure 4). Similarly, Taeseon showed a 19% increased transpiration rate at 7DAT compared to the control (Figure 4). In the case of *g*_*sw*_, Mallikong and Somyongkong showed no difference in Si treatment compared to the control for all data collection periods (Figure 4). Contrarily, *g*_*sw*_ significantly increased in Geomjeongsaeol (38%) and Taeseon (44%) after Si application compared to the control plants. Specifically, Geomjeongsaeol showed *g*_*sw*_ in Si treatment at 1DAT (41%) and 7DAT (38%), whereas Taeseon only showed significantly increased *g*_*sw*_ at 7DAT (Figure 4).

#### Experiment II: Distinguish Root Morphological Traits Among Contrasting Cultivars

**FIGURE 5:**
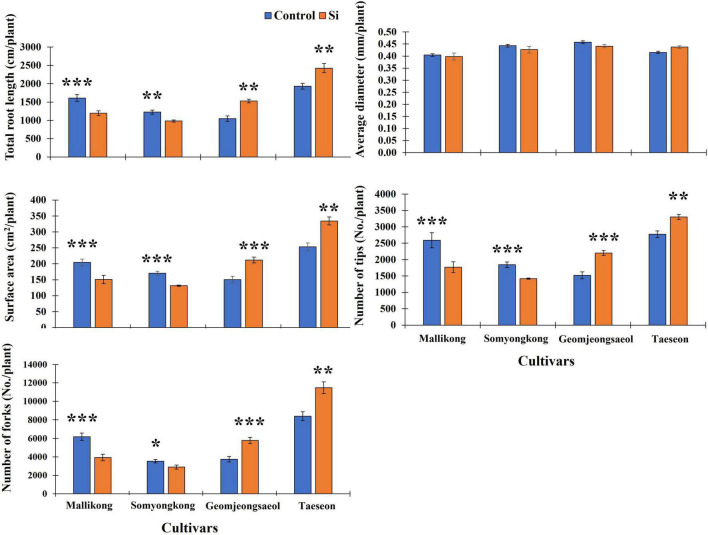
Silicon effect on root morphological and architectural traits in selected soybean cultivars. All data were collected in 7 days after Si treatment. In the figure, each blue and orange bar represents control and Si treatment, respectively, and the vertical bar with error bars indicate average ± standard error (*n* = *5*). The experiment was conducted by five replications. In the figure, asterisks indicate significant difference by Student’s *t*-test (*0.01 < *P* < 0.05; **0.001 < *P* < 0.01; ***P < 0.0001).

Using selected soybeans, we again measured the RMT of the selected plant samples with more numbers than those in EP-I. Thus, we observed distinguished RMTs between contrasting soybean cultivars (Figure 2). In the case of RMTs, TRL and SA showed a significant decrease in Si-treated Mallikong [approximately 24% (TRL) – 26% (SA)] and Somyongkong [approximately 17% (TRL) – 20% (SA)], whereas both the root traits significantly increased in Si-treated Geomjeongsaeol [approximately 46% (TRL) and 50% (SA)] and Taeseon [approximately 26% (TRL) and 34% (SA)] compared to the control (Figure 5). However, our results showed no difference in AD between the control and Si treatment, and this tendency was also detected in the whole soybean cultivars (Figure 5). We measured the related parameters to determine the Si effect on the RSA traits. According to our result, the obtained RSA, NT, and NF revealed significant differences in responses among contrasting cultivars (Figure 5). Si-treated Geomjeongsaeol and Taeseon showed increased NT (Geomjeongsaeol: 34% and Taeseon: 15% approximately) and NF (Geomjeongsaeol: 55% and Taeseon: 40% approximately), respectively (Figure 5). Somyongkong and Mallinkong revealed significantly decreased NT (Somyongkong: 18%, Mallinkong: 32%) and NF (Somyongkong: 9%, Mallikong: 36%) in the Si treatment compared to the control (Figure 5).

#### Experiment II: Relationship Between Various Root, Shoot, and Photosynthesis-Related Parameters

**FIGURE 6:**
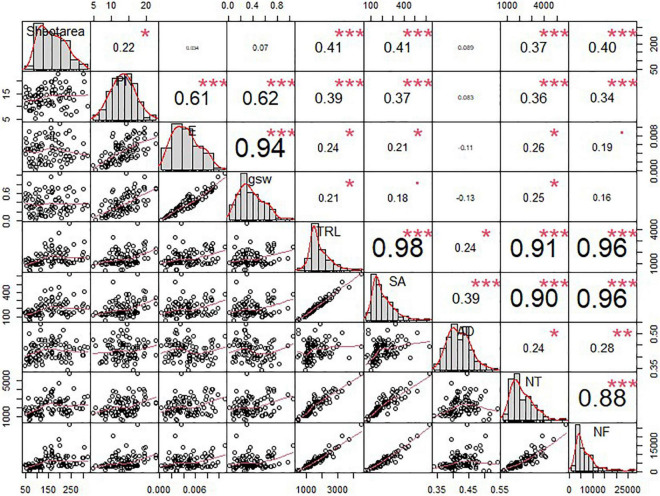
Correlation analysis among the shoot and root traits of soybean cultivars. Error bars indicate standard error, and the asterisks indicate significant difference using the correlation test (*0.01 < *P* < 0.05; **0.001 < *P* < 0.01; ***P < 0.0001).

Using various shoot and root phenotypes, we conducted a correlation test. To begin with, we analyzed the correlation between the shoot and root phenotypes. According to Figure 6, the shoot area revealed a significantly positive correlation with TRL (*r* = 0.41^∗∗∗^), SA (*r* = 0.41^∗∗∗^), NT (*r* = 0.37^∗∗∗^), and NF (*r* = 0.40^∗∗∗^). *P*_*N*_ also showed a positive correlation with TRL (*r* = 0.39^∗∗∗^), SA (*r* = 0.37^∗∗∗^), NT (*r* = 0.36^∗∗∗^), and NF (*r* = 0.34^∗∗∗^). Although *E* and *g*_*sw*_ also showed a positive correlation with root traits, the value was extremely low. In comparison among the shoot traits, the shoot area only showed a positive correlation with *P*_*N*_ (*r* = 0.22^∗^); however, its value was too low (Figure 6). A comparison between RMT (TRL, SA, and AD) and RSA traits (NT and NF) showed a significant correlation with each other. TRL showed a significantly positive correlation with NT (*r* = 0.91^∗∗∗^) and NF (*r* = 0.96^∗∗∗^); furthermore, the SA showed a higher positive correlation with NT (*r* = 0.90^∗∗∗^) and NF (*r* = 0.96^∗∗∗^) (Figure 6). In the case of AD, although AD revealed a positive correlation with NT (*r* = 0.24^∗^) and NF (*r* = 0.28^∗∗^) statistically, each value was lower than the other RMT (Figure 6). Conclusively, major shoot phenotypes, such as shoot area and *P*_*N*_, not only showed a positive correlation with RMT but also showed a positive correlation with the RSA traits (Figure 6).

#### Experiment II: Silicon Content of Two Soybean Cultivars

**FIGURE 7:**
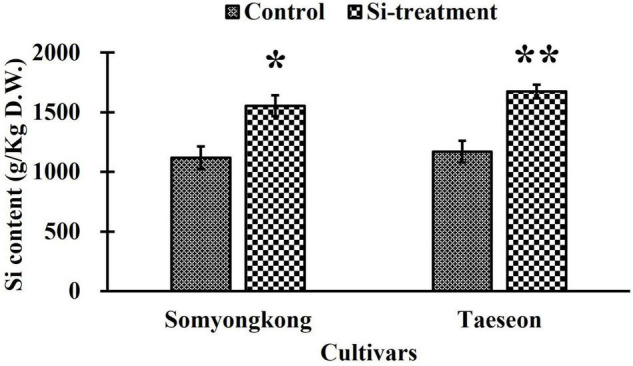
Comparison of Si accumulation in contrasting soybean cultivars after Si treatment. Error bars indicate standard error, and the asterisks indicate significant differences using the *T*- test (*0.01 < *P* < 0.05; **0.001 < *P* < 0.01).

Through EP-II, we identified distinct shoot and root phenotypes in contrasting soybean cultivars after Si treatment. Thus, we selected Somyongkong and Taeseon based on the results of EP-I and EP-II as materials in ICP analysis and gene expression associated with root growth and development. According to Figure 7, both the soybean cultivars exhibited significantly increased Si content in Si application compared to the control. In Somyongkong, the Si content was approximately 28.1% higher in the Si treated plant than in the control plant (Figure 7). Similarly, Taeseon showed approximately 31.1% increased Si content in the Si-treated plant compared to the control (Figure 7). Therefore, both contrasting cultivars frequently showed significantly elevated Si due to Si supplementation.

#### Experiment II: Gene Expression Analysis

**FIGURE 8:**
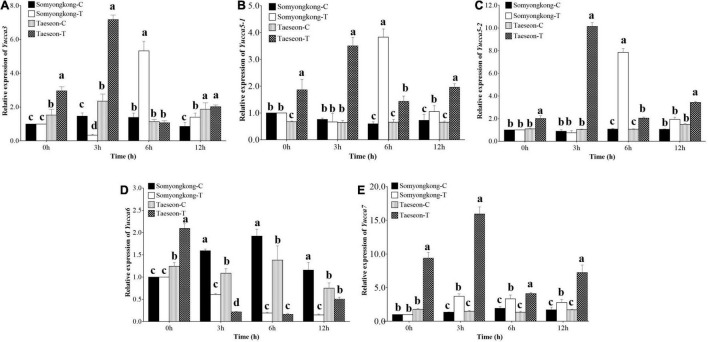
Relative expression levels of root development and auxin biosynthesis pathway genes. Expression levels of *YUC3*, **(A)** *YUC5-1*, **(B)** *YUC5-2*, **(C)** *YUC6*, **(D)** *YUC7*, and **(E)**, genes in the roots of control (C) and Si-treated (T) Somyongkong and Taeseon cultivars at different periods. All the values are mean ± SE (*n* = 3). Error bars indicate a standard error and different letters indicate the significant difference (*P* < 0.05) between treatments using the *T*-test.

**FIGURE 9:**
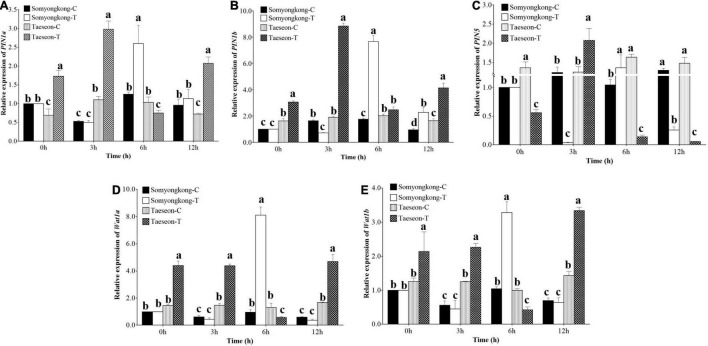
Relative expression levels of root auxin transporter genes. Expression levels of *PIN1a*, **(A)** *PINb*, **(B)** *PIN5*, **(C)** *WAT1a*, **(D)** *WAT1b*, and **(E)** genes in the roots of control (C) and Si-treated (T) Somyongkong and Taeseon cultivars at different periods. All values are mean ± SE (*n* = 3). Error bars indicate a standard error and different letters indicate the significant differences (*P*** <** 0.05) between treatments using the *T*-test.

According to EP-I and EP-II results, the positive response of the cultivar, Taeseon had the most improved root traits (TRL, SA, NT, and NF) after the Si treatment, whereas Somyongkong had negative responses for root traits (TRL, SA, NT, and NF) after the Si treatment. To further understand the molecular mechanisms underlying the positive and negative responses of the stated cultivars, we examined the expression levels of genes associated with root development and auxin transporter pathways (Ganguly et al., 2010; Wang et al., 2019) using two contrasting soybean cultivars (Figures 8, 9). The tryptophan aminotransferase of Arabidopsis (TAA)/YUCCA (*YUC*) gene expression level was significantly higher at 3 h (7DAT) in Taeseon cultivar compared to the control as well as the treated plants of Somyongkong (Figures 8A–C,E). However, the highest expression level was observed at 6 h (7DAT) in the Si-treated cultivar Somyongkong for *YUC3* and *YUC5-1*genes compared to the control and Si-treated Taeseon cultivar (Figures 8A,B). These results reveal the differential activation of *YUC* genes over the time point in two contrasting cultivars. Overall, the transcript accumulation of *YUC* genes was significantly higher in Si-treated Taeseon compared to Somyongkong.

Similarly, we examined the expression level of the transporter genes acting in auxin efflux and cell-wall formation [PIN-FORMED (*PIN*) and *WALLS ARE THIN* (*WAT*)], which also play a significant role in root hair growth. The expression levels of *PIN1a* and *PIN1b* genes were significantly higher in Si-treated Taeseon cultivar at 0, 3, and 12 h (7DAT) compared to both the control and Si-treated plants of Somyongkong cultivar (Figures 9A,B). Whereas *PIN5* gene expression level was significantly lower at 0, 6, and 12 h (7DAT) in Si-treated Taeseon cultivar compared to the control plants as well as the Somyongkong cultivar (Figure 9C). In addition, there was no difference observed between the control and the Si-treated Somyongkong cultivar at 0 h for all the *PIN* genes (Figures 9A–C). Interestingly, we observed that only at 6 h (7DAT), all the *PIN* (*PIN1a*, *PIN1b*, and *PIN5*) gene expression level was significantly higher in Si-treated Somyongkong cultivar compared to Taeseon (Figures 9A–C). The *WAT1a* and *Wat1b* gene expression levels were significantly higher in Si-treated Taeseon cultivar compared to the control as well as the Si-treated Somyongkong plants, at all-time points except at 6 h (Figures 9D,E). Overall, the expression level of genes related to root development, auxin transporter, or cell development pathways suggested higher expression patterns in Taeseon cultivar compared to the Somyongkong cultivar that may have caused a positive response for RMT in Taeseon.

## Discussion

The application of Si as a fertilizer is vital for economical and sustainable crop cultivation due to its beneficial effects, such as an increased number of tillers in rice and enhanced resistance to abiotic stress in rice (Ning et al., 2014). However, most of its benefits have been observed in monocotyledon plants, such as rice, wheat, and barley (Kim et al., 2014, 2017). According to Hodson et al. (2005), several dicotyledon plants also accumulate high concentrations of Si. Furthermore, Hodson et al. (2005) reported that the relative shoot Si concentration is 1.399, which ranks 610 among 735 plant species. Therefore, soybean could be regarded as high-Si accumulating crop. We conducted our previous study based on this background and discovered that soybean production increased significantly with Si fertilizer treatment than in the control plants (Tripathi D. K. et al., 2021; Tripathi P. et al., 2021). Moreover, Si fertilizer application significantly improves RMT. Although Si fertilizers include a higher concentration of Si, the previous study does not prove that only Si induces increased RMT and productivity. Si fertilizers contain not only Si (25%) but also include Mg (2%) and Ca (40%). For this reason, we used only Si in this study to obtain more accurate and confirmative results for determining the effect of Si on various shoots and RMT in soybean (*n* = 21) cultivars. Based on a previous study by Park et al. (2019), 2 mM Si was used in this study. Overall, the Si was uptake by the roots from the soil and transported to the shoot area with steam transpiration (Mitani and Ma, 2005; Yamaji and Ma, 2009). Recently, Deshmukh et al. (2020) have shown the relationship between soil Si and uptake in plants. The Si can be uptake by the stomata of leaves; however, only a minimal amount of Si can be absorbed with water. First, the root encounters Si among the plant organs (Tripathi P. et al., 2021). Thus, our study focused on the effect of Si on several root traits, and we obtained substantial results in soybean roots.

Overall, the concentration-dependent response of Si is widely observed in crops, including, soybean (Park et al., 2019), rice (Kim et al., 2014), Indian mustard (Rehman et al., 2016), and sugarcane (Frazão et al., 2020). However, the cultivar-dependent response of Si in soybean has not yet been identified. In line with our previous findings, variable cultivar-dependent responses to inorganic nutrients have been recorded in potatoes, and this phenomenon was induced by different availability (Chea et al., 2021). In addition, Bokor et al. (2019) group in demonstrated how Si modulates the root morphology in date palm. In this study, Si application enhanced the root traits; however, few soybean cultivars showed reduced root traits. Therefore, we selected contrasting soybean cultivars based on root features to determine the differences caused by Si treatment. We assessed the shoot area of four soybean varieties and observed variance in the shoot area. The shoot area was significantly reduced in Mallikong and Somyongkong cultivars to Si treatment, while Geomjeongsaeol and Taeseon showed a neutral response to Si application but showed no difference between the control and the Si-treated plants. Overall, the leaf area determines light interception, making it one of the crucial factors for photosynthesis prediction (Weraduwage et al., 2015). Thus, different shoot area frequently causes a distinction between the photosynthesis rate of plants. For this reason, we measured net-photosynthesis rate, transpiration rate, and stomatal conductance for 7 days. In the shoot area, Mallikong and Somyongkong showed a reduced tendency; however, *P*_*N*_, *E*, and *g*_*sw*_ showed no reduction after Si application. This pattern was detected in 3DAT, 5DAT, and 7DAT. Conversely, Geomjeongsaeol and Taeseon showed increased *P*_*N*_, *E*, and *g*_*sw*_ in Si treatment, although the shoot area showed no difference between the control and Si treatment. Conclusively, Si application caused a decline in the shoot area in Mallikong and Somyongkong, indicating a negative response to Si treatment, but these phenomena did not result in photosynthesis reduction. Therefore, this result indicated that photosynthesis rate or related parameters did not contribute to the reduction in the shoot area. A similar result was reported by Ruppenthal et al. (2016). They assessed leaf area after applying different amounts of Si (50, 100, 150, and 200 mg kg-1) to soybean (*Glycine max* L. cv. V-Max). Although the soybean plant grows well in water, the leaf area was reduced after Si application, and the rate of loss increased with higher Si treatment content.

From previous studies, we confirmed that Si treatment not only increases the shoot phenotype but also enhances the RMT (Guo et al., 2006; Chung et al., 2020; Tripathi D. K. et al., 2021). However, in our study, only a few soybean cultivars showed negative reactions to Si treatment, implying that differences in Si sensitivity among soybean cultivars could result in root phenotypic differentiation. Hence, we measured the RMT and RSA traits and compared the differences between contrasting cultivars. In different cultivars, the results differed. The TRL and SA considerably increased in Geomjeongsaeol and Taeseon when Si was applied; both cultivars exhibited a positive response to Si application compared to the control. Whereas TRL and SA of the other two cultivars, Mallikong and Somyongkong, considerably decreased in the Si treatment compared to the control plants. Other RMT, such as AD, did not exhibit any difference between Si treatment and control, and the same trend was observed in the entire soybean cultivars. In the 2D image, SA was influenced by two factors, TRL and SA. Therefore, if the SA of the plant increases, this phenomenon would have to ensure that both TRL and AD increase, or at least one of them. According to our findings, TRL fluctuation influences SA variation in response to exogenous Si treatment. However, Si application showed no effect on AD in soybean plants, although soybean plants showed contrasting responses to Si treatment. We examined differential reactions in RSA traits based on various responses to Si treatment. The cultivars, Geomjeongsaeol and Taeseon exhibited significant increases in RSA traits, NT, and NF, but Mallikong and Somyongkong displayed significant decreases. Hence, our result suggests that the effect of Si application on major RMT, except for AD and RSA traits, varies by cultivar. According to our results, Geomjeongsaeol and Taeseon showed positive responses, while Mallikong and Somyongkong revealed negative responses when Si was applied to the root zone and foliar area. Specifically, Si treatment resulted in a significant increase in root length, NT, and NF; consequently, SA also increased. However, according to our previous study, the application of Si fertilizer not only increases the nodule number but also enhances several root phenotypic attributes (Tripathi P. et al., 2021). The root diameter significantly increased by Si fertilizer treatment, while root length showed no difference statistically. We assumed that this limitation was caused by several reasons, such as (i) growth period for root sample collection, (ii) different methods of root sample collection, and (iii) additional effect of inorganic nutrients, such as Mg and Ca. In the previous experiment, we collected the root samples on the harvesting date; therefore, we dug out root samples with a shovel. Consequently, it was challenging to collect the whole root samples from soil; therefore root length could be affected due to loss of root particles. In this study, we collected relatively entire root samples because PVC pipe was used for the pot. Hence, we assumed that this difference caused differential results of root length between the two experiments. Furthermore, the Si fertilizer used in the previous experiment contained 2% of MgO and 40% of CaO (Tripathi P. et al., 2021). According to Niu et al. (2014), Mg concentration contributed to root hair development in Arabidopsis by regulating reactive oxygen and cytosolic Ca^2+^ concentration. Also, Murillo-Amador et al. (2007) reported that the application of Ca-silicate enhances resistance to salinity stress in two legume species by improving the root biomass. Therefore, our previous result was inherent in the effect of Mg and Ca on root phenotype. However, those concerns were removed by this experiment. Pure Si application can induce SA variation by reinforcing TRL, NT, and NF.

We conducted a correlation test and discovered that each shoot and root phenotype had a higher correlation. Shoot area showed a higher correlation with RMT, such as TRL, SA, NT, and NF, than shoot phenotypes related to photosynthesis. This result indicated that RMT and RSA traits acted as major factors in the formation of shoot area than photosynthesis-related parameters in this experiment. In the *P*_*N*_ and related parameters, *P*_*N*_ showed a higher correlation with RMT and RAT, but not AD. Therefore, this result assumed that most RMT and RSA traits not only affected the shoot area formation but also induced *P*_*N*_ and *E* activation. Moreover, we observed a significant correlation value between RMT and RSA traits, but not AD.

Through a series of evaluations, we discovered that (i) soybean plants showed a different response to Si treatment. (ii) Si treatment could enhance RMT and RSA traits. However, we did not know why Si induced a different response in RMT and RSA traits. To explain this question, we conducted an additional experiment. Among contrasting cultivars, we selected one from each side; thus only two cultivars, which were Somyongkong (negative response) and Taeseon (positive response). Using two contrasting soybeans, we conducted another experiment. All environmental conditions and Si application methods were similar to EP-II. Plant samples were harvested 7 days after Si application to analyze the Si content. According to the results, the Si content in the shoot part significantly increased in both cultivars; therefore, this result could be regarded as evidence for the upcoming phenomenon. Based on various root phenotypes, we assumed that the different RMT and RSA traits caused by Si application would contribute to gene regulation in root growth and development (auxin pathways). Auxin is one of the major plant hormones known to be involved in root growth and development. According to several reports, *YUC* genes are significantly involved in auxin biosynthesis; thus the absence of *YUC* genes leads to defects in root development (Mashiguchi et al., 2011; Stepanova et al., 2011). Besides, researchers suggested that *YUC* genes are mostly involved in the growth of the meristematic zone, transition zone, whereas *PIN* genes are involved in the growth of lateral root-forming zone and growth terminating zone (Qin and Huang, 2018). For this background, we analyzed the expression levels of some *YUC* genes in contrasting cultivars. Overall, we discovered that *YUC* gene expression in Taeseon was substantially higher compared to the control as well as Si-treated Somyongkong plants. This tendency was consistently observed at 0 and 3 h after Si treatment (7DAT). The expression results indicate that *YUC* gene activation differs depending on the time point in the two distinct cultivars. It also suggests that Si application could have induced variation in *YUC* gene transcript accumulation in contrasting cultivars. Similarly, the expression levels of *PIN* and *WAT* genes were investigated as they are involved in auxin transporter pathways, efflux, cell wall formation, and root hair growth. Results suggested that all *PIN* (*PIN1a*, *PIN1b*, and *PIN5)* gene expression levels were differentially regulated at different time points in the Si-treated Taeseon cultivar compared to the control as well as Si-treated Somyongkong cultivar plants (Figures 9A–C). Recently, Li et al. (2019) reported the function of *PIN1a* and *PIN1b* in rice; mutations in *PIN1a* and *PIN1b* affect root gravitropism, primary root length, plant height, crown root number, and lateral root number. Whereas *WAT1a* and *WAT1b* gene expression levels were significantly higher in Si-treated Taeseon cultivar compared to the control as well as Si-treated Somyongkong cultivar; over all time points, except at 6 h (Figures 9D,E). As far as we know, there is a lack of direct studies on the topic of Si-mediated molecular regulation of *PIN* and the possible roles of *YUCCA* gene in the root cultivar/genotype-dependent response.

Overall, higher expression patterns in Taeseon cultivar compared to the Somyongkong cultivar revealed stronger expression patterns in root growth, auxin transporter, or cell development pathways, which may have generated a good response to RMT in Taeseon. Previously, it has been shown that Si has a role in the regulation of critical genes involved in water transport, polyamine production, transcription regulation, defense, photosynthesis, and housekeeping during abiotic and biotic stresses (Manivannan and Ahn, 2017). Furthermore, several genes implicated in stress-response pathways, such as the phenylpropanoid pathway, antioxidant, and phytohormones, such as abscisic acid, ethylene, and jasmonic acid, are regulated by Si in a wide range of plant species (Yin et al., 2016; Manivannan and Ahn, 2017). Although Si plays a significant role in regulating the physiological, biochemical, and oxidative activities of the plant, the genetic basis of variation in root growth remains elusive. As a result, it is appealing to infer that the Si influences auxin and root development and transport pathway-related genes that consequently impact root growth and produce cultivar-dependent variation in soybean. However, this may be further subjected to a detailed molecular level investigation depending on the plant species.

## Conclusion

From this study, we discovered the interesting effects of Si on soybean plants. First, soybean cultivars showed a diverse response to root growth and development. Specifically, some soybean cultivars showed reduced TRL, SA, NT, and NF after Si treatment than the control. In our experiment, Somyongkong and Mallikong observed a negative effect on RMT and RSA traits. Contrarily, many soybean cultivars showed increased RMT and RSA traits in Si application compared to the control. To confirm different responses with soybean cultivars, we performed additional experiments. Contrasting soybean cultivars revealed that different expression levels of *YUC*, *PIN*, and *WAT* genes are involved in auxin biosynthesis, efflux of auxin from a cell, and auxin transporter pathways. In our experiment, the expression levels of *YUC*, *PIN*, and *WAT* genes were relatively upregulated in Taeseon compared to Somyongkong; therefore, we proposed that (i) Si treatment induced upregulation of auxin biosynthesis and transporter genes; thus auxin content and its transport were increased in soybean plant, which showed a positive response in Si treatment; (ii) increased auxin caused RMT and RSA trait improvement; (iii) improved RMT and RSA trait induced active *E* and *P*_*N*;_ (iv) these differential responses induced different phenotypic attributes in soybean. Additional investigations, such as an assessment of auxin content with and without Si treatment, are necessary to apply this concept in the negative response of soybean.

## Data Availability Statement

The raw data supporting the conclusions of this article will be made available by the authors, without undue reservation.

## Author Contributions

PT prepared a draft of the manuscript and analyzed the shoot and root phenotype data. RT was involved in the data analysis and improving and editing the manuscript. B-GM and B-WY assisted with the genetic data collection. YK inspected the experimental design and revised the manuscript. All authors contributed to the article and approved the submitted version.

## Conflict of Interest

The authors declare that the research was conducted in the absence of any commercial or financial relationships that could be construed as a potential conflict of interest.

## Publisher’s Note

All claims expressed in this article are solely those of the authors and do not necessarily represent those of their affiliated organizations, or those of the publisher, the editors and the reviewers. Any product that may be evaluated in this article, or claim that may be made by its manufacturer, is not guaranteed or endorsed by the publisher.

## Supplementary Material

Supplementary Figure 1 The flow chart of data analysis in the root sample using the WinRHIZO Pro software.
