# Supplementary figures and images for: Silicon Application Differentially Modulates Root Morphology and Expression of PIN and YUCCA Family Genes in Soybean (Glycine max L.)
Source: Front Plant Sci. 2022 Mar 18;13:842832. doi: 10.3389/fpls.2022.842832 (PMC8975267; doi:10.3389/fpls.2022.842832)

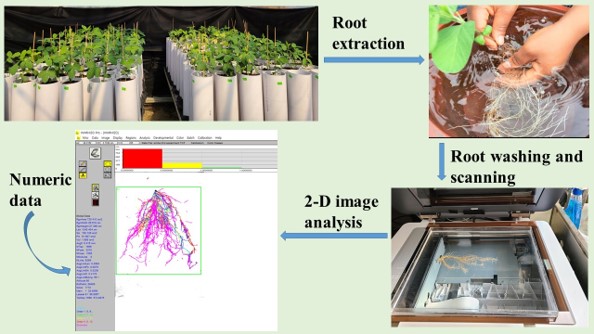

Supplement: Supplementary Figure 1 — The flow chart of data analysis in the root sample using the WinRHIZO Pro software. [file Image_1.jpg]
