# Supplementary material for: Silicon Application Differentially Modulates Root Morphology and Expression of PIN and YUCCA Family Genes in Soybean (Glycine max L.)
Source: Front Plant Sci. 2022 Mar 18;13:842832. doi: 10.3389/fpls.2022.842832 (PMC8975267; doi:10.3389/fpls.2022.842832)
Supplement: Supplementary file 2 [file Data_Sheet_1.docx]

**Table S1.** List of soybean cultivars used during this study

| IT number | Name | IT number | Name |
| --- | --- | --- | --- |
| IT 142810 | Baekunkong | IT 213176 | Saebyeolkong |
| IT 157893 | Hwasongputkong | IT 213194 | Geomjeongsaeol |
| IT 163461 | Williams 82 | IT 214696 | Daepoong |
| IT 180160 | Eunhakong | IT 214708 | Chungja 3 |
| IT 189209 | Mallikong | IT 229414 | Alchankong |
| IT 189210 | Gwangankong | IT 242605 | Hwangkeumkong |
| IT 203575 | Ilpumgeomjungkong | IT 263165 | Joongmo 3006 |
| IT 203580 | Ilmikong | IT 263182 | Taeseon |
| IT 211812 | Somyongkong | IT 270003 | Joongmo 3009 |
| IT 212859 | Daewonkong | IT 270006 | Jinpung |
| IT 213174 | Daechekong |  |  |

**Table S2.** Specification of camera used for shoot phenotyping

|  | Specification |
| --- | --- |
| Dimensions (H × W × D) | 108.2 × 67.1 × 35.1 mm |
| Weight | 302 g (including battery and memory card) |
| Image sensor | 22.3 × 14.9 mm CMOS (24.2 MP) |
| Image processor | DIGIC 7 |
| Lens | EF-M 11–22 mm F4-5.6 IS STM |

Note: The same methods with EP-I was employed in EP-II to collect the data of root phenotype.

**Table S3.** List of the primers used for expression analysis in this study

| Gene ID | Gene Name | Forword | Reverse |
| --- | --- | --- | --- |
| *AT3G18780* | *Actine* | GCTGGACGTGACCTTACTGA | CCATCTCCTGCTCGTAGTCA |
| *Glyma.10G128700* | *YUCCA3* | GCGAAAACTCGGAGAAAGTG | ATCCAGTGAGACCTCCATGC |
| *Glyma.10G041800* | *YUCCA5-1* | AGGTGTTTGGGAAGTCAACG | TGTCCAAAACAGGGGTCTTC |
| *Glyma.13G128800* | *YUCCA5-2* | ACAGTTCTGCCAGCTCCCTA | ACCGGCAAATGTACTCGAAC |
| *Glyma.06G081300* | *YUCCA6-like* | AACCCCTGTGCTAGATGTGG | CATTTGGAAATGGCCTCCTA |
| *Glyma.20G080000* | *YUCCA7* | GAGAGTTTGGTGGCCATGTT | ACCGTTTCATCAACGTCACA |
| *Glyma.06G111900* | *WAT1a-At4g08290* | ACCATTGGGTCATAGGGACA | GTTCTGCGAAGATTGCAACA |
| *Glyma.06G310700* | *WAT1b-At5g07050* | GGACAATTGGCTGGGATATG | GAGCCCATGATGGCTACAAT |
| *Glyma.08G054700* | *PIN1a* | GGAGTGGACCATAACGCTGT | GAGACAATGGTACCGGCAGT |
| *Glyma.07G102500* | *PIN1b* | CCTAGCTGCTGACACCCTTC | ACTGGAGGACCACAATTTGC |
| *Glyma.09G251600* | *PIN5a* | ATCGTTGTAGGATTGCGAGG | TCATACCAAAGATCACCGCA |
